# The effects of 3,5-diiodothyronine on energy balance

**DOI:** 10.3389/fphys.2014.00528

**Published:** 2015-01-13

**Authors:** Fernando Goglia

**Affiliations:** Dipartimento di Scienze e Tecnologie, Università degli Studi del SannioBenevento, Italy

**Keywords:** thyroid hormone, resting metabolism, 3-5-diiodothyronine, mitochondria, energy balance

## Introduction

Thyroid hormones (THs) have been known to affect energy metabolism (calorigenic effect) for over a century (Magnus-Levy, 1895; Thompson et al., 1929). In 1985 Magnus-Levy observed that patients with mixedema exhibited an abnormal low oxygen consumption when compared to normal individuals and that unusually higher amount of oxygen was consumed by hyperthyroid patients. 3,3′,5-triiodo-L-thyronine (T_3_) is the active form of THs and it is a major regulator of growth and development and of cellular and tissue metabolism (both intermediate and energy metabolism) throughout the body. Metabolic actions include regulation of: basal metabolic rate in homeotherms, synthesis of mitochondrial respiratory enzymes and membranes, oxidative phosphorylation and energy transduction, movement of water and Na^+^ ions across cell membranes; calcium and phosphorus metabolism, lipids synthesis and storage, catabolism of fatty acids, cholesterol, carbohydrate; and nitrogen (urea, creatine) metabolism; growth and developmental actions include actions on: rate of postnatal growth of many mammalian and avian tissue, maturation of fetal brain and bone, amphibian larval metamorphosis, and molting in birds. It is now recognized that T_3_ affects gene expression in target tissues/cells by binding to its cognate nuclear receptors (TR) which are ligand-inducible transcription factors. Two TR genes α and β encode four T3-binding receptor isoforms (α1, β1, β2, and β3). The transcriptional activity of TRs is regulated at multiple levels. Besides being regulated by T3, transcriptional activity is also regulated: (i) by the type of thyroid hormone response elements located on the promoters of T3 target genes, (ii) by the developmental- and tissue-dependent expression of TR isoforms, and (iii) by a host of nuclear coregulatory proteins (corepressors and coactivators). These nuclear proteins modulate the transcription activity of TRs in a T3-dependent manner. In the absence of T3, corepressors act to repress the basal transcriptional activity, whereas in the presence of T3, coactivators act to activate transcription. The activities regulated via the previous described mechanisms are described as “genomic actions.” However, between the mid-1980's and the beginning of the 1990's it became evident that some TH effects are non-genomic in origin. Indeed, high-affinity binding sites for thyroid hormones have for many years been recognized on the plasma membrane and other cellular sites such as mitochondria and cytoplasm (for review see Cheng et al., 2010). Recently, a structural protein of the plasma membrane, integrin αvβ3, has been shown to contain a binding domain for iodothyronines that is an initiation site for hormone-directed complex cellular events, such as cell division and angiogenesis (Bergh et al., 2005) and this qualifies the binding site for characterization as a receptor. Examples of non-genomic action of thyroid hormones are activation of: membrane Ca2-ATPase activity, 2-Deoxyglucose transport, Na, K-ATPase activity, Na^+^ current in myocardiocytes, Na^+^ current in sensory neuron, Na^+^/H^+^ exchanger, cancer cell proliferation, angiogenesis (for review see Cheng et al., 2010). In addition to this, it is now recognized that other iodothyronines or THs analogs/derivatives are able to exert relevant biological actions (for recent review, see Moreno et al., 2008; Senese et al., 2014; Zucchi et al., 2014). This article is particularly intended to describe the effects of the 3,5 diiodo-L-thyronine (T_2_) on energy balance (Moreno et al., 1997; Goglia, 2005).

## 3,5-diiodo-L-thyronine (T_2_)

**Figure 1:**
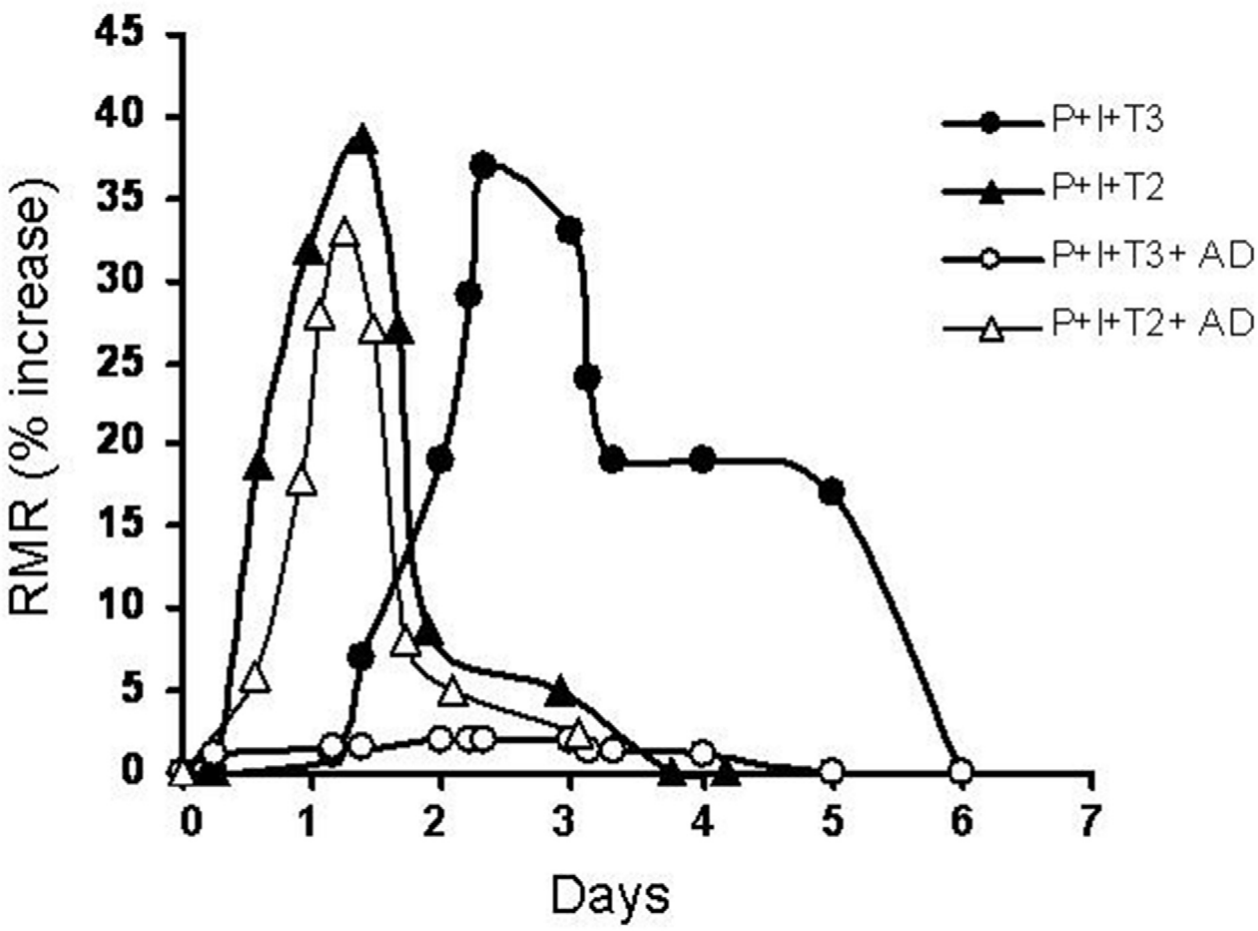
**Time course of variation in the resting metabolic rate of hypothyroid rats following administration of a single dose of iodothyronines (25 μg/100 g BW for both T_2_ and T_3_) with or without a concomitant administration of actinomycin D (8 μg/100 g BW) (AD)**. Hypothyroidism was induced by combined treatment with PTU and IOP (P + I). Resting metabolic rate (RMR) is reported as % increase [vs. time 0 (immediately before the injection)].

T_2_, a naturally occurring diiodothyronine, is a product of a currently unknown enzymatic process most probably utilizing T3 as its precursor (Moreno et al., 2002). Some years ago surprising results were published showing that (among a lot of iodothyronines tested) T_2_, at a very low concentration (pM), induced a rapid stimulation of oxygen consumption in perfused livers isolated from hypothyroid rats. In the same study, it was shown that T_3_ showed a similar effect but this effect was largely abolished by the addition of an inhibitor of D1 deiodinase, while the effect of T_2_ was not. Moreover, T_2_ exerted its effect more rapidly than T_3_ (Horst et al., 1989). Stimulated by that report and another study showing an interaction of a diiodothyronine with mitochondria (Goglia et al., 1981) some laboratories started to investigate more deeply on possible specific biological actions of T_2_. Initially, energy metabolism was the major area of interest. Indeed, several reports from various laboratories showed that acute or chronic administration of T_2_ to rats resulted in significant changes in energy metabolism. When either T_3_ or T_2_ were acutely injected to hypothyroid rats, T_2_ had a more rapid effect on resting metabolic rate than T_3_ (Lanni et al., 1996). The experimental design used in this study was basically the same as that employed by Tata in the early 1960's (Tata et al., 1962; Tata, 1963) and the only difference was that in the study of Lanni et al. hypothyroidism was achieved by the simultaneous administration of propylthiouracil (PTU) and iopanoic acid (IOP). This treatment produces animals with severe hypothyroidism and at the same time, with a powerful inhibition of all three types of deiodinase enzymes. In such conditions the effects of T_2_ were evident as soon as 1 h after its injection reaching the maximal value after 24 h, while that of T_3_ became evident only after 24 h reaching the maximal value after 72 h (these effects of T_3_ were overlapping to those obtained by Tata, 1963). Moreover, while the effect of T_3_ was inhibited by a inhibitor of transcription such as actinomycin D (as shown also by Tata, 1963). the effect of T_2_ was not (Lanni et al., 1996; Moreno et al., 1997) (see Figure 1).

Following these results studies continued to try to clarify the mechanisms underlying the previous described results. In light of the effects of T2 on energy metabolism, the mitochondria became the obvious candidates to study such mechanisms. In this context, some years ago, by top-down elasticity analysis, it was showed a stimulation of the activity of both cytochrome c-oxidizers and the cytochrome c-reducers components of the respiratory chain, 1 h after the injection of 3,5-T2 (Lombardi et al., 1998). These data indicate a possible direct interaction of T2 with some components of the respiratory chain. Indeed, this hypothesis was in agreement with previous results showing a direct stimulation of the enzyme cytochrome oxidase (COX) activity isolated from bovine heart (Goglia et al., 1994). Arnold and Kadenbach (1997) showed that (in addition to the mitochondrial membrane potential, the substrate pressure in the respiratory chain and the oxygen concentration) the respiration of animal cells is also controlled by the matrix ATP/ADP ratio, via an interaction of nucleotides with COX. In fact, ATP produces an allosteric inhibition of the COX activity. In a further investigation, Arnold et al. (Arnold et al., 1998) showed that 3,5-T2 specifically binds to subunit Va of the COX complex and completely abolishes the allosteric inhibition of respiration induced by ATP. Subunit Va of the COX complex is therefore a mitochondrial site through which T_2_ may directly affect mitochondrial activities. In addition to activating mitochondrial substrate oxidation, 3,5-T_2_ also stimulates skeletal muscle mitochondrial uncoupling in a very rapid manner (Lombardi et al., 2007, 2009). By discriminating between proton-leak and redox-slip processes, an increased mitochondrial proton conductance has been addressed as the “pathway” underlying the effect of T2 on mitochondrial uncoupling. Thus, activation of COX complex above described associated to changes in the efficiency of the skeletal muscle mitochondrial energy-transduction apparatus, may explain, at least in part, the rapid effect of T2 on metabolic rate.

The stimulatory effects exerted by T_2_ on RMR prompted my group to investigate on a possible effect of this iodothyronine in counteracting overweight and lipid accumulation without deleterious side effects in particular on hearth and skeletal muscle such as those showed by T_3_ when tested as slimming agent. To test this idea, we administered T2 for 30 days to rats on a high fat diet (HFD) and then we measured the adipose tissue mass, the body weight gain, the liver adiposity, the liver fatty acid oxidation rate, and the serum levels of triglyceride, fatty acids, and cholesterol. In this study we also looked at a possible effect of T_2_ on HPT axis (Lanni et al., 2005). The results showed that, except a slight decrease (−20%) in T_4_ serum level, no variation in the Hypothalamus-Pituitary-Thyroid axis (HPT) was evident measured by the “TRH-test.” In this study rats treated with T2 showed lower body weight, a higher liver fatty acid oxidation rate, less fat mass, an almost complete disappearance of fat from the liver, and significant reductions in the serum triglyceride and cholesterol levels. Recently, most of these results have been confirmed also in animals with a standard laboratory diet and with a prolonged time of treatment (Padron et al., 2014). In addition, several studies from both our and others laboratories showed relevant biological effects of T_2_ and some of them reported beneficial effects of T_2_, among others:

Moreno et al. (2011) and de Lange et al. (2011) showed that T_2_ prevented high-fat-diet-induced insulin resistance in rat whose action involved activation of sirtuin 1 (SIRT1).
Markova et al. (2013) showed an antidepressant-like effect of T2 in rats after a bolus administration of T2 at the doses 75 and 150 μg/100 g b.w.
Shang et al. (2013) showed that T2 was a protective agent against renal damage in diabetic nephropathy in streptozotocin-induced diabetic rats, confirming the involvement of sirtuin 1 (SIRT1).

These effects were observed in absence of deleterious side effects. However, some studies have shown some deleterious effect at cardiac level and on HPT axis (Goldberg et al., 2012; Jonas et al., 2014) but in these studies unusual very high doses of T_2_ were studied and unspecific interaction of T_2_ with nuclear TRs may have occurred.

Further details about the actions of T_2_ can be found in some reviews (Goglia, 2005; Coppola et al., 2014; Senese et al., 2014).

Of Note, the understanding of the physiological and pathophysiological role of T2 would benefit from development and standardization of new methods for analytical measurement of T_2_ and other TH derivatives. Actually, measurements of T2 in human tissue and sera have been so far taken using immunoassays (Kirkegaard et al., 1981; Nishikawa et al., 1981). However, the lack of labeled iodothyronines with high specific activity as well as of specific antibodies has represented an important limitation to the application of such approach. Promising developments in this field have recently emerged from the optimization of a new competitive chemiluminescence immunoassay (CLIA) based on the use of one selected mouse monoclonal anti-T2 antibody with very low cross-reactivity to structurally related THs and thyronamines (Lehmphul et al., 2014). Mass spectrometry techniques have also drawn attention to the analyses of iodothyronines (Köhrle et al., 2013), however, intrinsic instrumental limits still restrain the application of such approaches as routine tools.

## Conclusion and perspectives

The data reported and discussed in this article have generated a large interest in the possibility of identifying analogs/derivatives of thyroid hormone that may prove effective as therapeutic agents to counteract some major diseases that are growing in importance worldwide. But, before this perspective can be realized further studies are needed to elucitade the cellular/molecular mechanisms of action of these agents and, in addition, a possible therapeutic use of these agents need deep investigations on possible deleterious side effects especially when administered for a long time. As for T_2_ it remains to be established whether it has a physiological role or not and in an affirmative case it remains unclear what are the possible physiological roles that differentiate the actions of T2 and T3. Another aspect is related to the problem of possible TR-mediated genomic effect of T_2_. It has been recently proven that T_2_ is a specific ligand for a long isoform of TRβ in tilapia (Mendoza et al., 2013) affecting the growing processes in this specie. T3 and T2 both participate in the growth process, however their effects are mediated by different, specific TRβ1 isoforms (STRβ_1_ and LTRβ_1_ respectively). However, no data are present at the moment showing that a such binding can affect energy metabolism in higher species and further studies are needed to verify this possibility. Further studies may also be useful to try to develop new concepts that could help toward a better understanding of some of the effects of thyroid hormones and those of their analogs/derivatives.

### Conflict of interest statement

The author declares that the research was conducted in the absence of any commercial or financial relationships that could be construed as a potential conflict of interest.
